# Child malnutrition crisis in Gaza: a humanitarian and public health emergency

**DOI:** 10.3389/fpubh.2025.1719762

**Published:** 2025-11-24

**Authors:** José Francisco López-Gil

**Affiliations:** 1 School of Medicine, Universidad Espíritu Santo, Samborondón, Ecuador; 2 Vicerrectoría de Investigación y Postgrado, Universidad de Los Lagos, Osorno, Chile

**Keywords:** child malnutrition, Gaza, public health, humanitarian crisis, conflict settings

According to United Nations Children's Fund, more than 7,000 children under 5 years of age in Gaza were admitted to recovery programs for acute malnutrition within 2 weeks in August, with over 15,000 cases expected for the month (more than seven times the number reported in February) (1, 2). Famine conditions have now been confirmed in Gaza City, with warnings that they could spread further south (3). This unprecedented escalation reflects the combined impact of conflict, displacement, and restricted humanitarian access (3, 4).

Child malnutrition in conflict settings is both immediately life-threatening and developmentally devastating (5). Severe wasting weakens the immune system and leaves children up to 11 times more likely to die from common infections (5, 6). Beyond survival, evidence demonstrates that early childhood undernutrition impairs cognitive development, lowers school achievement, and reduces adult economic productivity (5). These effects are often irreversible, contributing to intergenerational cycles of poor health and socioeconomic disadvantage.

Scientific literature shows that survivors of severe acute malnutrition experience persistent deficits in growth, cognition, and behavior (5). Women who were malnourished as children are more likely to have low-birth-weight infants, transmitting vulnerability to the next generation (5). In Gaza, health workers warn that today's malnourished infants face permanent impacts on their health and development (2). Without urgent action, the legacy of this crisis will be measured not only in lives lost now but also in the diminished potential of thousands of children.

Fortunately, severe malnutrition is preventable and treatable. Ready-to-use therapeutic foods (RUTF) have a recovery rate of around 90% when treatment is initiated early (3, 6). Scaling up RUTF distribution, therapeutic milks, vaccination, and treatment of infections is essential. Psychosocial stimulation should also be integrated into nutritional programs. World Health Organization recommends this to improve cognitive and developmental outcomes in children recovering from severe malnutrition (7). Strengthening community-based screening and management is equally critical to identify and treat cases before children deteriorate. Humanitarian agencies continue to face severe logistical and political barriers in delivering essential aid to Gaza. Blockades, damaged infrastructure, and restrictions on the entry of food, fuel, and medical supplies have further worsened the crisis, undermining timely access to lifesaving interventions (4).

The crisis in Gaza represents a humanitarian and public health emergency. We urge the global health community to advocate for unimpeded humanitarian access and the rapid expansion of evidence-based nutritional and psychosocial interventions. The right to adequate nutrition and survival is fundamental. Gaza's children deserve nothing less.
